# Generalizability and validation considerations in AI-guided novice focused cardiac ultrasound

**DOI:** 10.1093/ehjdh/ztag081

**Published:** 2026-05-28

**Authors:** Liam Munir, Adele Cai, April Cai

**Affiliations:** School of Medicine and Dentistry, Griffith University, 1 Parklands Drive, Southport, 4215 Gold Coast, Australia; School of Medicine and Dentistry, Griffith University, 1 Parklands Drive, Southport, 4215 Gold Coast, Australia; School of Medicine, Newcastle University, Newcastle upon Tyne NE1 7RU, United Kingdom

Kane and colleagues^1^ report an evaluation of AI-guided focused cardiac ultrasound (FoCUS) performed by truly inexperienced operators, demonstrating that with 4 h of training a two-step workflow (AI interpretation, with cardiologist overread of abnormal or uninterpretable scans) achieved a sensitivity of 96.2% and specificity of 95.4% for the detection of left ventricular ejection fraction (LVEF) <40%. The technical achievement is meaningful, and the imaging protocol and reader blinding are well documented. We raise two concerns that we believe materially affect how these findings can be applied to the screening role the authors propose.

Our first concern relates to threshold selection and the role of the validation cohort. The ‘optimal threshold’ for the AI classifier was selected in the primary 496-patient cohort to maximize sensitivity for LVEF<40%, and the two-step workflow was constructed from that derivation. The subsequent 344-patient cohort, presented as validation, used the same threshold and workflow at the same single institution with the same three operators using the same equipment. This is internal performance confirmation rather than external validation. The reported validation sensitivity of 100% rests on six events (95% confidence interval 70.1–100.0), and a point estimate based on this number of events cannot anchor the inferential weight currently placed on it in the conclusion.^2^ We would welcome clarification on whether the threshold and workflow were pre-specified before the validation cohort was recruited, and would value performance reporting at thresholds that external groups would be likely to apply.

Our second concern is the inferential gap between paired diagnostic accuracy and screening utility. The cohort comprised patients referred for diagnostic echocardiography, with the FoCUS examination performed within 30 min of the reference echo. Indications were predominantly symptomatic or surveillance-driven: known or suspected valvular disease (22.9%), known or suspected arrhythmia (20.5%), and known or suspected cardiomyopathy or heart failure (16.5%). This is a paired diagnostic test in a pre-selected, indication-defined population, not a screening evaluation. The case mix differs from a screening population in ways that prevalence alone does not capture, including the distribution of ECG abnormalities, body habitus, comorbidity, and the prior probability of subclinical structural disease, all of which interact with image quality and AI calibration.^3,4^ Applying the reported sensitivity and specificity to community LVEF <40% prevalence estimates of 1–2%^5,6^ yields the PPV range shown in *Table 1*: between 71% and 83% of ‘positive’ novice scans in a community deployment would be false positives requiring confirmatory echocardiography. The authors note that ‘a lower prevalence of disease in a healthier screening cohort may yield different results,’ but the screening framing in the abstract, graphical abstract, and conclusion (which describes the technology as holding ‘promise for facilitating a framework for low-cost community screening’) is not directly supported by the data presented, even accounting for that caveat.

**Table 1:** Recalculated positive and negative predictive values of the two-step FoCUS workflow (sensitivity 96.2%, specificity 95.4%) at varying disease prevalence

Disease prevalence	Positive predictive value	Negative predictive value	Setting
1%	17.4%	99.96%	Unselected community screening (lower bound)
2%	29.9%	99.92%	Unselected community screening (upper bound)
5.5%	54.9%	99.77%	Kane *et al*. study cohort
10%	69.9%	99.56%	High-risk primary care population

A third observation: the proposed workflow targets LVEF <40% only. The authors acknowledge that Stage B HFpEF was not assessed. We would add that an AI-guided FoCUS framework limited to severe LV systolic dysfunction is best characterized as a tool for HFrEF identification rather than for cardiac dysfunction more broadly, given that HFpEF represents over half of incident heart failure in older populations^7^ and forms the dominant phenotype in the screening-relevant age band. Significant valvular disease, infiltrative cardiomyopathy, and hypertrophic cardiomyopathy are also missed by an LVEF-centric threshold. Aligning the framing of the target condition with what the test actually detects would help readers situate the technology within the broader screening agenda.

None of these observations diminishes the achievement of demonstrating that 4 h of training plus AI guidance can yield diagnostically usable images in 95% of patients referred to a tertiary echocardiography service. Our concern is narrower: the conclusion that the technology may serve as a framework for community screening requires (i) external validation at a different site with different operators and a pre-specified threshold, (ii) recruitment that does not select for the indication-defined case mix of an echocardiography laboratory, and (iii) explicit framing of the target condition as HFrEF rather than cardiac dysfunction. We look forward to the authors’ response and to subsequent work in cohorts representative of the populations in which this technology might ultimately be deployed.
